# Gender Differences in Determinants of the Components of the Frailty Phenotype among Older Adults in India: Findings from LASI Wave-1

**DOI:** 10.3390/ijerph20043055

**Published:** 2023-02-09

**Authors:** Sayani Das, Jitender Prasad

**Affiliations:** 1 International Institute of Health Management Research, Delhi 110075, India; 2 International Institute for Population Sciences, Mumbai 400088, India

**Keywords:** community-dwelling, frailty phenotype, handgrip strength, older people, physical activity

## Abstract

This study examines the gender-specific determinants of the components of frailty in a community-dwelling setting in India. Using data from the Longitudinal Ageing Study in India (LASI) Wave-1, this study employed 30,978 (14,885 male and 16,093 female) older adults (aged 60+) to fulfil the study objective. The modified Fried frailty phenotype criteria defines frailty by the five components: exhaustion, weak grip strength, slow walking speed, unintentional weight loss, and low physical activity. The result showed grip strength (79.1%) as the most discriminant component among males, and physical activity (81.6%) as the most discriminant component among females. The results also indicated that grip strength (male: 98.0%, female: 93.5%) and physical activity (male: 94.8%, female: 96.9%) showed a sensitivity of more than 90%, which appears to be a good indicator of frailty. Combining this dual marker increased the accuracy to 99.97% among male and 99.98% among female samples. The findings suggested adding grip strength and physical activity as a proxy measure of frailty, which can increase the precision of screening without a large additional investment of time, training, or cost.

## 1. Introduction

Physical frailty in aging is an emergent state of a dysregulated complex dynamical system [1], it is also an important phenotype used to understand the vulnerable nature of older adults [2]. Physical frailty can be considered as pre-disability, where disability is defined as the need for assistance with basic activities of daily living [3]. Globally, a substantial amount of research has been conducted to understand frailty syndrome. Epidemiological evidence shows that about 3.9% to 51.4% of older adults experience physical frailty in community-dwelling older adults in low-income and middle-income countries, as defined by the Fried phenotype criteria [4]. In India, little is known about the nature of frailty syndrome in community-dwelling older adults (aged 60-year-olds and above). However, most previous efforts to study frailty in the country have been restricted to non-representative samples in certain geographical regions. The largest and most comprehensive study on frailty syndrome in India (WHO-SAGE 2007) explained that India has the highest number of cases of frailty among the six low and middle-income countries (China, Ghana, Mexico, Russia, and South Africa) [5]. They also highlighted that the gap between the gender-specific prevalence of frailty syndrome was wider for India. The national prevalence of physical frailty for males was 27.4% and 32.2% for females according to the Longitudinal Aging Study in India (LASI), 2017–2018 [6]. In India, the aging population is a socially unequal and heterogeneous section with a male and female divide. For the first time, the Census of India 2001 pointed out the feminization of the aging population, which continues to show the trend of more aged females than males, since the last Census of India conducted in 2011 [7]. Females are more likely to live longer than males, so they are more prone to have a relatively inferior health status during their old age [8]. Several studies have highlighted that females tend to experience physical frailty more than males [9,10,11]. Therefore, it is necessary to begin treating frailty in males and females differently in community-dwelling older adults in India.

Highlighting the impending aging population in India’s forthcoming decades, it is essential to understand the onset of physical frailty. The early detection of frailty syndrome is vital to prevent complications that otherwise burden health and social care systems. It is also essential for the intervention of the physical frailty components that are first detected, while reversal may still be possible [12]. However, a major difficulty in studying frailty syndrome is the absence of a gold standard measuring scale. An extensive review of 545 articles on frailty assessment instruments reported 67 methods for screening frailty syndrome [13]. They also explained that the frailty phenotype scale is the most commonly used tool for assessing risk factors to find out the biomarkers of frailty, prevalence estimation, inclusion–exclusion criteria, and as a guide for decision-making and intervention. To the best of our knowledge, no studies have assessed the gender-specific determinants of the components of the Fried frailty phenotype in India. It is important to determine which components of the frailty phenotype are the most informative to establish frailty in the community-dwelling population. We hypothesized that each component might present a specific and different screening ability, which may help determine frailty more efficiently. Therefore, this study examines gender differences in determinants of the Fried frailty phenotype components among older adults in a community-dwelling setting in the second-largest populated country (India).

## 2. Materials and Methods

### 2.1. Data Source and Study Sample

The Longitudinal Aging Study in India (LASI) Wave-1 (2017–18), a nationally representative data, was used in this study. The LASI collected data from 73,396 people aged 45 and over, as well as their spouses (of any age), across India’s states and union territories. This data resulted from a collaborative survey between the Harvard T.H. Chan School of Public Health, the International Institute for Population Sciences (IIPS), and the University of Southern California (USC). LASI data were collected based on internationally comparable research designs, instruments, and cutting-edge scientific procedures. This data offered the foundation for trustworthy and acceptable statistics—for national and state-level policymakers, as well as a long-term scientific study. The data collected information related to demographics, household economic status, family and social networks, work and employment, retirement, chronic health conditions, functional health, symptom-based health conditions, mental health, biomarkers, health insurance and healthcare utilization, welfare programs, and life satisfaction. The sampling was based on multistage stratified cluster sample design, which includes three and four separate phases of rural and urban region selection [14]. More detailed information about the survey design is available at https://www.iipsindia.ac.in/lasi (accessed on 2 October 2022). The present study investigates a total sample size of 30,978 (14,885 older males and 16,093 older females) after dropping the missing cases for physical frailty aged 60-year-olds and above.

### 2.2. Assessment of Frailty

Frailty was evaluated using the modified Fried frailty phenotype scale [9]. This screening tool consists of five physiological deficits:

- Exhaustion: Individuals were asked two questions from the CES-D scale: “how often they felt tired or low in energy last week?”. Those who responded “often” (3 or 4 days) or “most or all of the time” (5–7 days) were considered Exhausted = 1, and others were considered as No = 0.
- Weak grip strength: LASI measured handgrip strength using a handheld Smedley’s Hand Dynamometer. The average score (in kg) for the two successive trails of the dominant hand were calculated and considered as the final grip strength. The score in the bottom quintile was observed as weak grip strength and was adjusted for gender and body mass index. Low grip strength was considered as Yes = 1, and other was considered as No = 0.
- Slow walking time: Respondents were asked to walk 4 m at a usual walking pace twice. The time taken to walk was recorded in seconds, and the mean time was calculated. The score in the bottom quintile of the time values was adjusted for gender and height (median). Slow walk was considered as Yes = 1, and other was No = 0.
- Unintentional weight loss: Individuals were asked “whether they thought they had lost weight in the last 12 months because there was not enough food in their household”. Those who responded Yes = 1 were considered as having weight loss, and others were considered as No = 0.
- Low physical activity: We did not calculate physical activity as assessed by Fried et al., (2001) using the Minnesota Leisure Activity Questionnaire [9]. In LASI, participants were asked about their physical activity “How often do you take part in sports or vigorous activities”. Those who did not engage in moderate or vigorous physical activity (1–3 times a month or hardly ever or never) for a given time throughout the week were considered Physically Inactive = 1, and others were considered as Active = 0.

Dichotomous variables were created for all of the five symptoms, and a frailty score was generated by summing all of the scores. The total physical frailty phenotype score was between 0 and 5. For this study, all samples were classified as “frail” if they met 3 or more of the 5 criteria, and below 3 were classified as “non-frail”. A number of research works have validated this modified frailty phenotype scale by using slightly different cut-off points [15,16,17].

### 2.3. Background Characteristics

This study considered individual-level potential predictors, including age (60–69 years, 70–79 years and 80+ years), sex (male and female), educational status (no education, primary, secondary, and higher and above), marital status (currently in wedlock and not in wedlock not in wedlock includes, respondents who were widowed/separated/divorced/never married), place of residence (rural and urban), work status (never worked, not working, working, and retired). Other potential behavioural and health-related variables consist of tobacco use (no and yes representing ever use of tobacco products) and alcohol use (no and yes, representing ever drinking alcohol). Activities of daily living (ADL) refers to normal daily self-care activities of the study participants, such as movement in bed, changing position from sitting to standing, feeding, bathing, dressing, grooming, and personal hygiene. Combining these ADLs, a single variable was generated. It was categorized as “no” if the respondent did not face difficulty performing any ADLs and “yes” if they faced difficulty performing one or more ADLs. In the instrumental ADL (IADL), respondents were asked if they were having any difficulties that were expected to last more than three months, preparing a hot meal, shopping for groceries, making a telephone call, taking medications, doing work around the house or garden, managing money (such as paying bills and keeping track of expenses), and getting around or finding an address in unfamiliar places. Combining these IADLs, a single variable was generated. It was categorized as “no” if the respondent did not face difficulty performing any IADLs and “yes” if they faced difficulty performing one or more IADLs.

### 2.4. Statistical Analysis

Descriptive statistics were used to summarize (age and gender-wise) and to provide a background characteristic of the study population. The significance level of the bivariate model was tested using Fisher’s chi-square test (χ2 test). Age and gender differences of the frailty components were determined in the study samples, and the national sample weight was used for computing the percentage distribution. Sensitivity, specificity, positive predictive value, negative predictive value, and prevalence were calculated for single-component and dual-component markers by constructing 2 × 2 contingency tables. Standard epidemiologic definitions were used for the calculations. The 95% CIs were calculated using the Wilson procedure with continuity correction [18]. All statistical analyses were performed using STATA (version 16.1, StataCorp LLC, College Station, TX, USA) software.

## 3. Results

Table 1 presents the age-group and gender-specific background characteristic of the study participants. The final sample comprised 48.1% males and 51.9% females. Irrespective of gender, it was observed that the majority of participants were residing in rural areas (male: 67.1%, female: 65.4%). About 88.2% of females aged 80 years and above were currently not in wedlock, which was higher compared with the male participants (42.5%) in the same age category (*p* < 0.001). The results also showed that 9.0% of males aged 80 years and above had higher education and above, while only 0.86% did among the female participants (*p* < 0.001). Irrespective of age group, smoking (*p* < 0.001) and alcohol (*p* < 0.001) consumption were found to be significantly higher among the older male participants, while ADL and IADL factors were higher among the female participants (*p* < 0.001).

Table 2 shows the prevalence of Fried frailty phenotype components by age group and gender in India. The overall prevalence of frailty was 27.9% for male and 33.2% for female participants. It was found that each frailty component ranged from 5.9% (weight loss) to 79.1% (grip strength) among the male participants and 5.5% (weight loss) to 81.6% (physical activity) among the female participants. In males, the most prevalent component was grip strength (72.3%, 86.8%, and 95.3%), and the lowest was weight loss (5.8%, 5.9%, and 6.2%). However, females also had weight loss (5.2%, 6.3%, and 5.1%) as the lowest prevalent component, while the highest was physical activity (76.9%, 87.1%, and 93.0%).

**Figure 1:**
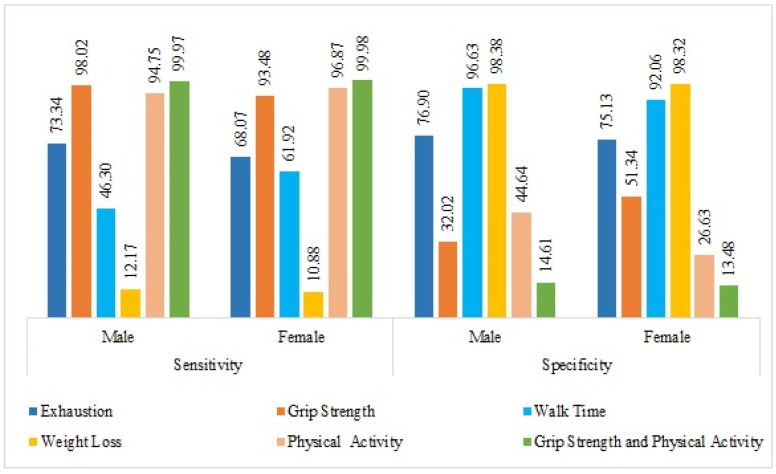
Sensitivity and specificity of the frailty phenotype components by gender.

Table 3 presents the diagnostic accuracy of the Fried frailty phenotype components among male and female participants in India. Irrespective of gender, grip strength (male: 98.0%, female: 93.5%) and physical activity (male: 94.8%, female: 96.9%) showed a sensitivity of more than 90%, which appeared to be a good indicator of frailty in this study. The positive predictive value of the individual components for predicting frailty ranged from 36.2% to 84.0% among males, while it was found to be 38.5% to 80.7% in females. All possible dual-trait component combinations were performed in this sample (results not shown). Out of these, grip strength and physical activity together were found in more precise results. Combining this dual marker increased the sensitivity to 99.97% among male and 99.98% among female samples. Figure 1 presents the sensitivity and specificity of frailty phenotype components among older male and female participants.

## 4. Discussion

The results of our study, involving a large sample from the older Indian population, demonstrate that each of the Fried frailty phenotype scale components has a specific and different diagnostic ability for accessing baseline physical frailty in the community-dwelling population. From our findings, it is apparent that the use of grip strength in males and physical activity in females alone was sensitive and accurate as a proxy measure for the Fried frailty phenotype. The dual-component measure of grip strength with physical activity was more accurate than individual components in both males and females than other possible dual-component combinations.

A low grip strength was positively associated with aging [19], and several studies have shown a significant correlation between this parameter and various morpho-functional, social, and psychometric features [20]. Globally, it is already well documented that grip strength is an easy and cost-effective test, as well as a robust predictor of physical frailty [21,22,23,24,25]. A population-based frailty study in Brazil (FIBER) explored the reduction in handgrip strength as an initial manifestation of frailty, and found that may be present even before the emergence of other functional disabilities [26]. Most studies are based on the Western population, and not much data are available for the older Indian population in order to understand the association between grip strength and frailty syndrome among older adults. In India, Das and Chandel (2018) studied frailty patterns among older females in Haryana, Northern India [27]. They highlighted that weak hand grip strength is the fundamental feature of frailty among the study participants, as their study showed that most participants had a lower grip strength than the other four indicators. A previous study from LASI 2017–18 also emphasized that approximately 70.0% of older adults in India had a weak grip strength as a frailty marker [28]. However, our study extends the observations of previous studies to examine the gender-specific predictability of grip strength in order to understand frailty in India, and highlights it alone as a good indicator of frailty syndrome. In our study, it is also shown that grip strength showed the best sensitivity and accuracy for the male participants than the other frailty components. Generally, grip strength becomes weaker with age, eventually affecting older adults’ quality of life [29,30]. However, older males are more vulnerable because of dynapenia [31,32], as they exhibit more age-related loss of muscle strength [33,34]. Men with low testosterone levels have been shown to have low grip strength as a result of androgen deprivation [35]. A longitudinal study of Danish older adults also revealed a significantly greater change in grip strength in males compared with females in later life [36]. Therefore, our findings suggest using handgrip strength as a proxy measure of physical frailty, especially for community-dwelling male older adults. This study recommends routine evaluation of hand grip strength among older adults as it may have diagnostic and prognostic values. It is also inexpensive, easy to use, and non-invasive.

Our findings underscore the importance of physical activity in understanding physical frailty in the community-dwelling older population of India. It is essential to understand the intensity of daily physical activity of the geriatric population because empirical evidence has shown a very significant role of low physical activity status as the onset of the progression of physical frailty [37,38,39]. The Birjand Longitudinal Aging Study (BLAS) has stated that low physical activity is the most important determinant of physical frailty [40]. Fried (2016) illustrated that “physical activity offers the first evidence of effective approaches to preventing or treating frailty and a biologic model for future therapies” [41] (p. 11). Angulo and colleagues (2020) also stated physical activity as a strategy to manage frailty by acknowledging the association between these two factors [42]. They showed that physical activity helps the aging population reduce age-related oxidative damage and chronic inflammation. At the same time, it increases autophagy and improves mitochondrial function, myokine profile, insulin-like growth factor-1 (IGF-1) signaling pathway, and insulin sensitivity. Worldwide, lack of physical activity is considered the most important risk factor for frailty, especially the frailty phenotype [39,43,44,45], which is in line with our study. Previous studies have also highlighted that the relationship between physical activity and frailty syndrome persist in India [46,47]. However, the present study found that the association was too strong for female participants than for the other frailty components. Several sources indicate that females are more sedentary and inactive and less engaged in regular exercise and leisure time physical activity than males [48,49]. Lee (2005) also mentioned that the personal backgrounds of older females were less favorable for physical activity than those of males [50]. In the World Health Survey [51], the prevalence of physical inactivity in India was 9.3% in males and 15.2% in females. Therefore, the present study confirms the importance of female’s physical activity in old age to improve quality of life. Notably, exercise intervention programs and early detection can improve the hallmarks of frailty among older female adults in India.

Our study adds to the literature that combined markers of both grip strength and physical activity tools indicate the best baseline physical frailty in India for both males and females. Screening for all five frailty components within primary health care practice may be impractical, which can create barriers to widespread frailty screening in developing countries such as India, where a high percentage of older adults are living in rural areas. Therefore, as a proxy measure of the Fried frailty phenotype, we suggest adding grip strength and physical activity, which can increase the precision of screening without a large additional investment of time, training, or cost. To the best of our knowledge, this is the first study evaluating gender differences in the determinants of the frailty phenotype components in the community-dwelling population in India. This study has provided generalizable estimates using a nationally representative sample. The novel contribution of the study is that it included older adults from different socio-economic backgrounds and health conditions. The study also adopted the widely used and validated the Fried frailty phenotype criteria, which have been associated with adverse health outcomes in multiple conditions, therefore improving the application of research as well policy-making of the findings. There are some limitations to the current study that should be mentioned and addressed in future studies for a better determination of the components of the frailty in community-dwelling older adults in India, including that this study used a physical frailty phenotype scale, which limited the ability to understand bio-psycho-social frailty approach. In addition, India is an extremely heterogeneous country; the current observations underscore the necessity for a state-wise management strategy for the early detection and prevention of frailty in India.

## 5. Conclusions

In conclusion, regular access to physical activity status and handgrip strength in older adults serves as an easy, quick, and feasible screening instrument for physical frailty in the community-dwelling population. Gender-stratified analysis is useful for evaluating frailty, and it helps in the development of public health measures. Further studies are needed, however, in order to determine how effectively the better frailty components predict outcomes. The practical implications of the findings are connected with the health benefits of the community-dwelling population in India. It will help in the conceptualization of physical frailty and will help improve the quality of life of the population and reduce their future healthcare expenses.

## Figures and Tables

**Table 1:** Background characteristics of the study population (in percentage) in India, 2017–2018.

Background Characteristics (%)		Male					Female				
		60–69	70–79	≥80	N	*p*-Value	60–69	70–79	≥80	N	*p*-Value
**Place of Residence**	Urban	28.61	29.89	25.25	4892	<0.001	31.39	31.04	27.87	5572	0.071
	Rural	71.39	70.11	74.75	9993		68.61	68.96	72.13	10,521	
**Marital Status**	Currently in wedlock	86.71	77.10	57.53	12,246	<0.001	57.88	31.68	11.81	7513	<0.001
	Not in wedlock	13.29	22.90	42.47	2639		42.12	68.32	88.19	8580	
**Education level**	No education	35.07	40.03	46.71	5398	<0.001	68.58	74.74	82.81	11,230	<0.001
	Primary	28.26	30.44	32.85	4441		17.97	16.9	13.28	3012	
	Secondary	23.41	18.2	11.49	3240		9.39	6.11	3.04	1291	
	Higher and above	13.25	11.34	8.95	1806		4.06	2.25	0.86	560	
**Work Status**	Never worked	3.35	4.26	5.12	811	<0.001	45.84	50.1	52.02	7940	<0.001
	Currently working	56.77	30.89	12.22	6281		25.21	11.64	2.56	2951	
	Not currently working	29.45	50.05	68.63	5784		27.14	36.29	43.45	4846	
	Retired	10.43	14.8	14.03	1991		1.81	1.97	1.97	349	
**Alcohol**	No	69.45	73.78	79.22	10,227	<0.001	97.43	97.51	97.18	15,414	0.775
	Yes	30.55	26.22	20.78	4651		2.57	2.49	2.82	679	
**Smoking**	No	39.85	41.87	41.65	6549	<0.001	79.31	76.70	74.32	12,417	<0.001
	Yes	60.15	58.13	58.35	8327		20.69	23.30	25.68	3670	
**ADL**	No	85.29	78.57	63.03	12,323	<0.001	81.34	69.13	57.48	12,319	<0.001
	Yes	14.71	21.43	36.97	2559		18.66	30.87	42.52	3772	
**IADL**	No	69.99	57.26	40.51	9770	<0.001	52.01	37.66	24.75	7729	<0.001
	Yes	30.01	42.74	59.49	5100		47.99	62.34	75.25	8333	
**Total**		**47.04**	**48.86**	**47.50**	**14,885**	**<0.001**	**52.96**	**51.14**	**52.50**	**16,093**	**<0.001**

**Table 2:** Prevalence of frailty phenotype by age group and gender in India, 2017–2018.

Fried Frailty Component	60–69 Year Old		70–79 Year Old		80+ Year Old		Total	
	Male N (W %)	Female N (W %)	Male N (W %)	Female N (W %)	Male N (W %)	Female N (W %)	Male N (W %)	Female N (W %)
Exhaustion	3053 (37.04)	3675 (37.97)	1672 (39.26)	1820 (41.14)	616 (39.18)	717 (43.92)	5341 (37.94)	6212 (39.54)
Weak grip strength	5619 (72.3)	5023 (58.54)	3406 (86.84)	2998 (77.54)	1219 (95.32)	1183 (89.18)	10244 (79.07)	9204 (67.09)
Walk time	695 (8.06)	1572 (16.96)	816 (19.72)	1472 (35.43)	544 (45.23)	805 (58.45)	2055 (15.4)	3849 (26.31)
Weight loss	364 (5.76)	443 (5.22)	207 (5.90)	224 (6.32)	74 (6.20)	76 (5.10)	645 (5.85)	743 (5.53)
Low physical activity	5181 (55.68)	7640 (76.94)	3268 (73.14)	3843 (87.06)	1301 (85.85)	1538 (92.95)	9750 (64.21)	13021 (81.63)
**Frailty combined**	**1610** **(19.87)**	**2346** **(24.81)**	**1475** **(35.04)**	**1887** **(41.58)**	**764** **(51.34)**	**943** **(56.8)**	**3849** **(27.85)**	**5176** **(33.16)**

**Table 3:** Gender-specific diagnostic accuracy of the Fried frailty phenotype components in India, 2017–2018.

Fried Frailty Components	Sensitivity % (95% C.I.)	Specificity % (95% C.I.)	Positive Predictive Value (95% C.I.)	Negative Predictive Value (95% C.I.)	Prevalence (95% C.I.)
**Male**					
**Individual Marker**					
Exhaustion	73.34 (72.63,74.06)	76.9 (76.22,77.58)	52.65 (51.84,53.45)	89.18 (88.68,89.68)	25.93 (25.23,26.64)
Grip strength	98.02 (97.78,98.25)	32.02 (31.23,32.81)	36.17 (35.35,36.98)	97.62 (97.36,97.88)	28.21 (27.45,28.97)
Walk time	46.3 (45.46,47.14)	96.63 (96.32,96.93)	84.04 (83.42,84.66)	82.43 (81.79,83.08)	27.72 (26.96,28.47)
Weight loss	12.17 (11.64,12.69)	98.38 (98.17,98.58)	72.25 (71.53,72.97)	76.31 (75.63,77)	25.8 (25.09,26.5)
Physical activity	94.75 (94.39,95.11)	44.64 (43.84,45.44)	37.41 (36.63,38.18)	96.06 (95.74,96.37)	25.88 (25.18,26.58)
**Combined Markers**					
Grip strength and physical activity	99.97 (99.95,100)	14.61 (14.02,15.21)	31.28 (30.5,32.06)	99.93 (99.89,99.97)	27.99 (27.24,28.75)
**Female**					
**Individual Marker**					
Exhaustion	68.07 (67.35,68.79)	75.13 (74.46,75.8)	56.52 (55.75,57.29)	83.21 (82.63,83.78)	32.2 (31.48,32.92)
Grip strength	93.48 (93.07,93.88)	51.34 (50.52,52.16)	51.38 (50.56,52.2)	93.47 (93.06,93.87)	35.49 (34.7,36.27)
Walk time	61.92 (61.13,62.72)	92.06 (91.62,92.51)	80.75 (80.1,81.39)	81.81 (81.18,82.44)	34.96 (34.18,35.74)
Weight loss	10.88 (10.4,11.37)	98.32 (98.12,98.52)	75.37 (74.7,76.04)	70.03 (69.32,70.74)	32.07 (31.35,32.79)
Physical activity	96.87 (96.6,97.14)	26.63 (25.95,27.31)	38.51 (37.76,39.26)	94.72 (94.37,95.07)	32.17 (31.45,32.89)
**Combined Markers**					
Grip strength and physical activity	99.98 (99.96,100)	13.48 (12.92,14.03)	38.43 (37.64,39.22)	99.92 (99.88,99.97)	35.07 (34.3,35.85)

## Data Availability

The detailed methodology with complete survey design and data collection information is available at https://www.iipsindia.ac.in/lasi (accessed on 2 October 2022).
